# Risk of Obstructive Sleep Apnea in Patients with Emphysema Versus Bronchiectasis: A Propensity Score-Matched Cohort Study

**DOI:** 10.3390/healthcare14162659

**Published:** 2026-08-21

**Authors:** Ling-Hui Chang, Wen-Che Hsieh, Hui-Cheng Lin, Chao-Yu Hsu

**Affiliations:** 1 Division of Respiratory Therapy, Ditmanson Medical Foundation, Chia-Yi Christian Hospital, Chia-Yi 600566, Taiwan; 2 Department of Chinese Medicine, Ditmanson Medical Foundation, Chia-Yi Christian Hospital, Chia-Yi 600566, Taiwan; 3 Department of Nursing, Min-Hwei Junior College of Health Care Management, Tainan 73658, Taiwan; 4 Department of Physical Therapy, Lin Shin Medical Corporation, Lin Shin Hospital, Taichung 408346, Taiwan; 5 Department of Medical Education, Ditmanson Medical Foundation, Chia-Yi Christian Hospital, Chia-Yi 600566, Taiwan; 6 Department of Artificial Intelligence and Healthcare Management, Central Taiwan University of Science and Technology, Taichung 406053, Taiwan; 7 Department of General Education, National Chin-Yi University of Technology, Taichung 411030, Taiwan

**Keywords:** obstructive sleep apnea, emphysema, bronchiectasis, TriNetX

## Abstract

Background: Obstructive sleep apnea (OSA) is common in patients with chronic respiratory disease, but whether its risk differs between emphysema and bronchiectasis remains unclear. We compared the subsequent risk of OSA between these two structural lung diseases using a large real-world database. Methods: This retrospective cohort study used data from the TriNetX research network. Adults with emphysema or bronchiectasis were identified and followed for incident OSA. Baseline demographic characteristics, comorbidities, and medication use were balanced using 1:1 propensity score matching. OSA risk was evaluated at 1-, 2-, 3-, 4-, and 5-year intervals and cumulatively through 29 July 2026, using risk ratios, odds ratios, and hazard ratios (HRs). Results: Before matching, 526,877 patients with emphysema and 147,700 with bronchiectasis were included. After propensity score matching, 129,761 patients remained in each cohort, with all standardized differences below 0.10. In the matched analysis, OSA risk was modestly lower in the emphysema group at 1 year (HR = 0.829, 95% CI = 0.778–0.883), 2 years (0.885, 0.843–0.931), 3 years (0.923, 0.884–0.964), 4 years (0.946, 0.910–0.984), and 5 years (0.960, 0.925–0.995). However, the cumulative hazard did not differ significantly between groups (HR = 1.006, 95% CI = 0.976–1.038). Conclusion: After propensity score matching, bronchiectasis was associated with a modestly higher early risk of OSA, but this difference diminished over time and was absent over long-term follow-up. These findings should be considered hypothesis-generating and warrant prospective confirmation.

## 1. Introduction

Emphysema is a chronic structural lung disease characterized by destruction of the alveolar walls, enlargement of distal airspaces, and loss of elastic recoil. Although cigarette smoking remains its major risk factor, computed tomography (CT)-based studies have shown that emphysema can also occur in never-smokers, indicating that environmental exposures and host susceptibility may also contribute to its development [1,2]. From a physiological perspective, loss of elastic recoil promotes expiratory airflow limitation and air trapping, which can lead to lung hyperinflation and unfavorable changes in respiratory mechanics [3,4]. Hyperinflation may place the inspiratory muscles at a mechanical disadvantage and increase the work required to maintain ventilation [4]. These abnormalities may become particularly relevant during sleep, when ventilation and respiratory muscle activity normally decrease. In patients with obstructive lung disease, these physiological changes may aggravate hypoventilation, ventilation–perfusion mismatch, and nocturnal oxygen desaturation, thereby providing a plausible link between emphysema-related respiratory impairment and sleep-disordered breathing [5,6].

Bronchiectasis is also a chronic structural lung disease, but the mechanisms underlying respiratory impairment differ substantially from those of emphysema. Bronchiectasis is characterized by irreversible bronchial dilatation and is associated with impaired mucociliary clearance, persistent airway infection, and chronic inflammation, the structural airway damage interact in a self-perpetuating “vicious vortex,” thereby promoting progressive airway injury and impaired gas exchange, which interact to perpetuate airway and lung damage [7,8]. Sleep-related breathing disturbances have been increasingly recognized in patients with bronchiectasis. A recent systematic review and meta-analysis showed a significant prevalence of polysomnography-confirmed obstructive sleep apnea (OSA) in bronchiectasis patients but the data were from relatively small observational studies [9].

Both emphysema and bronchiectasis may predispose patients to sleep-related respiratory impairment, but through different underlying mechanisms. Emphysema is predominantly associated with loss of elastic recoil, alveolar destruction, gas trapping, and hyperinflation, whereas bronchiectasis is characterized mainly by airway dilatation, mucus retention, chronic infection, and inflammation [3,7,8]. Despite the potential relationship between these chronic pulmonary disorders and sleep-disordered breathing, direct evidence comparing the subsequent risk of OSA between patients with emphysema and those with bronchiectasis remains limited. Therefore, using a large real-world electronic health record (EHR) database, we compared the incidence and risk of OSA between patients with emphysema and those with bronchiectasis before and after propensity score matching. We hypothesized that the risk of OSA would differ between the two groups because of their distinct effects on airway function, respiratory mechanics, and gas exchange.

## 2. Materials and Methods

### 2.1. Data Source

This retrospective cohort study used data from the TriNetX research network, accessed on 29 July 2026. TriNetX is a global federated health research platform that includes 180 participating healthcare organizations and provides access to de-identified EHR data from approximately 190 million individuals. The database integrates demographic information, diagnoses, procedures, and medications results from participating institutions. All patient-level data within TriNetX are de-identified prior to inclusion in the network, in accordance with the Health Insurance Portability and Accountability Act (HIPAA) Privacy Rule. De-identification is attested through a formal determination by a qualified expert, ensuring that no protected health information is accessible to investigators. Consequently, analyses conducted using TriNetX data are generally considered exempt from institutional review board oversight.

### 2.2. Study Population

We identified adult patients aged 20 years or older who were newly diagnosed with emphysema or bronchiectasis between 1 January 2010, and 31 December 2021. Diagnoses were defined using International Classification of Diseases, Tenth Revision (ICD-10) codes recorded in the EHR. Patients were categorized into two cohorts according to their initial diagnosis: the emphysema (ICD-10: J43) cohort and the bronchiectasis (ICD-10: J47) cohort. The index date was defined as the first recorded diagnosis date of emphysema or bronchiectasis, respectively. To minimize potential confounding from pre-existing respiratory conditions, we excluded patients with a documented diagnosis of lung malignancy (ICD-10: C34) prior to the index date. In addition, patients with a history of OSA (ICD-10: G47.33) before the index date were excluded, given the potential impact of cancer-related factors on respiratory outcomes and sleep-related disorders. Patients with a diagnosis of bronchiectasis were excluded from the emphysema cohort, whereas patients with a diagnosis of emphysema were excluded from the bronchiectasis cohort to ensure that the two cohorts were mutually exclusive.

### 2.3. Covariates and Baseline Characteristics

Baseline demographic variables included age at index date and sex. Race was categorized as White and Black or African American, based on available EHR documentation. We also extracted information on clinically relevant comorbidities present before or at the index date. These comorbid conditions included dyslipidemia (ICD-10: E78), diabetes mellitus (DM) (ICD-10: E08-E13), ischemic heart disease (ICD-10: I20-I25), depression (ICD-10: F32), chronic kidney disease (CKD) (ICD-10: N18), and obesity (ICD-10: E66). Medication use before or at the index date was also recorded because pharmacologic exposure may influence sleep-related breathing disorders. The medications assessed included glucocorticoids (Anatomical Therapeutic Chemical [ATC] code: H02AB), statins (ATC code: C10AA), selective serotonin reuptake inhibitors (SSRIs) (ATC code: N06AB), metformin (ATC code: A10BA) and drugs used in nicotine dependence (ATC code: N07BA) including nicotine and varenicline.

### 2.4. Propensity Score Matching

To reduce selection bias and confounding due to baseline differences between the emphysema and bronchiectasis cohorts, we applied propensity score matching. Propensity score matching was performed in a 1:1 ratio, with scores generated directly within the TriNetX platform using built-in analytic tools. The propensity score model included age at the index date, sex, race, all prespecified comorbidities, and medication use. Balance between the matched cohorts was assessed using standardized differences (Std diff), with values less than 0.1 indicating acceptable covariate balance.

### 2.5. Outcome Assessment

The primary outcome was incident OSA diagnosed after the index date. OSA was identified using the corresponding ICD-10 code recorded in the EHR. Patients were followed from the index date until the first documented diagnosis of OSA, death, loss to follow-up, or the end of the observation period, whichever occurred first. The cumulative incidence of OSA was evaluated at 1, 2, 3, 4, and 5 years after the index date. An additional cumulative analysis through 29 July 2026, was conducted to assess the association over the longest available follow-up period.

### 2.6. Statistical Analysis

Comparative risk estimates between the emphysema and bronchiectasis cohorts were calculated using multiple complementary measures, including risk ratios (RRs) and odds ratios (ORs) with corresponding 95% confidence intervals (CIs). Time-to-event analyses were performed to estimate hazard ratios (HRs), accounting for variable follow-up durations. The proportional hazards assumption was evaluated using a proportionality test, with the corresponding *p* values reported for each follow-up period. All analyses were conducted within the TriNetX analytic environment, which reports aggregated, de-identified results only. Statistical significance was assessed and results were interpreted in conjunction with Std diff to account for the large sample size.

## 3. Results

**Figure 1:**
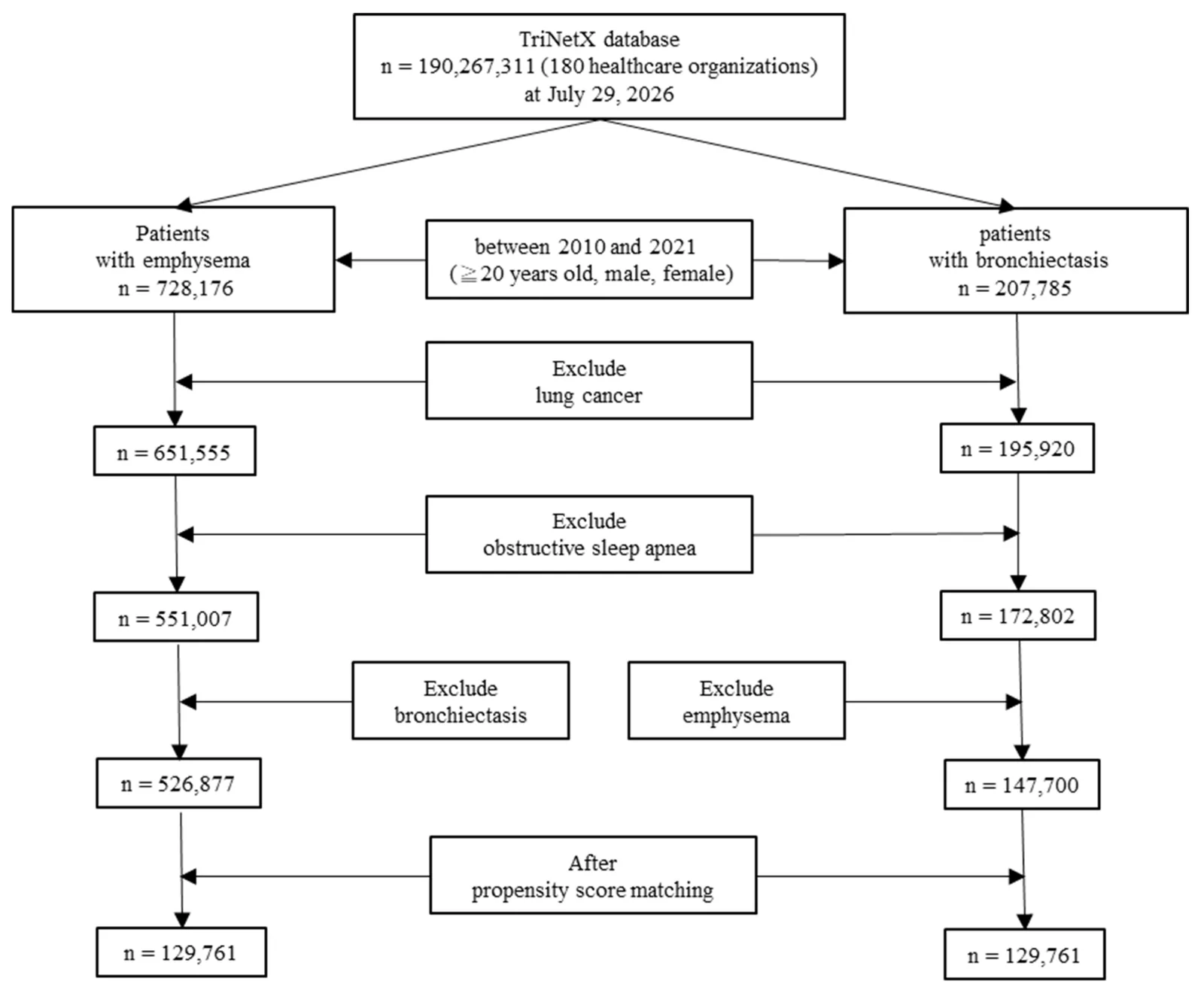
The flowchart of patients’ selection.

The flowchart illustrating patient selection is shown in Figure 1. Before propensity score matching, the study included 526,877 patients with emphysema and 147,700 patients with bronchiectasis (Table 1). Patients with emphysema were slightly older than those with bronchiectasis (64.0 ± 11.4 vs. 62.7 ± 14.7 years, *p* < 0.0001). The emphysema cohort had a lower proportion of women (44.92% vs. 60.84%) and a higher proportion of White patients (71.79% vs. 55.06%). Several comorbidities were more prevalent among patients with emphysema, including dyslipidemia (33.38% vs. 23.84%), ischemic heart disease (19.43% vs. 12.41%), depression (14.28% vs. 9.31%), DM (14.85% vs. 13.59%), CKD (7.94% vs. 7.22%), and obesity (8.50% vs. 6.85%). Medication use was also generally higher in the emphysema cohort, particularly statins (26.55% vs. 17.33%), SSRIs (12.66% vs. 8.72%), and drugs used in nicotine dependence (12.66% vs. 2.08%). All between-group comparisons were statistically significant (*p* < 0.0001), with notable baseline imbalances observed for sex, race, dyslipidemia, statin use, and nicotine-dependence medications.

After propensity score matching, 129,761 patients were included in each cohort, and the baseline characteristics were well balanced between the emphysema and bronchiectasis groups (Table 2). Patients with emphysema were slightly older than those with bronchiectasis (63.4 ± 13.6 vs. 62.6 ± 14.7 years, *p* < 0.0001), although the Std diff was small (0.0540). The proportions of female patients were nearly identical between the two groups (58.72% vs. 58.86%, *p* = 0.4655). Race distributions were also comparable, with only minimal differences in White and Black or African American patients. Comorbidities, including dyslipidemia, DM, ischemic heart disease, depression, CKD, and obesity, were similarly distributed between the matched cohorts. Medication use was also well balanced, including glucocorticoids, statins, SSRIs, metformin, and drugs used in nicotine dependence. Although several comparisons remained statistically significant because of the large sample size, all Std diffs were below 0.10, indicating adequate covariate balance after propensity score matching.

Before propensity score matching, the risk of OSA was consistently higher in patients with emphysema than in those with bronchiectasis, with the difference becoming more pronounced over longer follow-up (Table 3). At 1 year, the OSA risks were 1.544% and 1.492%, respectively, with no significant difference in hazard (HR = 1.033, 95% CI = 0.986–1.083). By 2 years, emphysema was associated with a modestly increased OSA risk (2.503% vs. 2.342%; HR = 1.071, 95% CI = 1.031–1.111). The association strengthened at 3, 4, and 5 years, with corresponding HRs of 1.119, 1.155, and 1.177. Cumulatively through 29 July 2026, OSA occurred in 36,266 patients with emphysema (6.883%) and 8703 patients with bronchiectasis (5.892%), yielding an RR of 1.168 (95% CI = 1.142–1.195), OR of 1.181 (95% CI = 1.152–1.209), and HR of 1.251 (95% CI = 1.222–1.281). Proportionality testing was nonsignificant at 1 and 2 years but significant thereafter.

After propensity score matching, the risk of OSA was generally lower in patients with emphysema than in those with bronchiectasis during the first 5 years of follow-up (Table 4). At 1 year, OSA occurred in 1.336% of patients with emphysema and 1.649% of those with bronchiectasis, corresponding to an HR of 0.829 (95% CI = 0.778–0.883). Similar reductions were observed at 2 years (2.226% vs. 2.586%; HR = 0.885, 95% CI = 0.843–0.931), 3 years (2.979% vs. 3.335%; HR = 0.923, 95% CI = 0.884–0.964), 4 years (3.688% vs. 4.051%; HR = 0.946, 95% CI = 0.910–0.984), and 5 years (4.313% vs. 4.701%; HR = 0.960, 95% CI = 0.925–0.995). Cumulatively through 29 July 2026, OSA was identified in 7784 patients with emphysema (5.999%) and 8435 with bronchiectasis (6.500%). Although the cumulative RR and OR remained below 1.0, the cumulative HR was 1.006 (95% CI = 0.976–1.038), indicating no significant overall difference in hazard. Proportionality testing was nonsignificant at 1 and 2 years but significant thereafter.

## 4. Discussion

In the present study, we compared the risk of OSA between patients with emphysema and those with bronchiectasis. After propensity score matching, no significant difference in the long-term hazard of OSA was observed between patients with emphysema and those with bronchiectasis. However, patients with bronchiectasis showed a modestly higher risk of OSA during the early follow-up period, and this difference gradually diminished over time.

The association between emphysema and OSA is most commonly discussed in the context of the chronic obstructive pulmonary disease (COPD)–OSA “overlap syndrome.” In patients with COPD, OSA does not appear to be universally more prevalent than in individuals without airflow limitation; however, when both conditions coexist, the combined physiological burden significantly worsens nocturnal oxygenation and clinical outcomes [10,11,12]. From a clinical perspective, overlap syndrome—regardless of the extent of emphysema—is consistently linked to greater cardiovascular comorbidity, more frequent COPD exacerbations, poorer quality of life, and increased healthcare utilization compared with either condition in isolation [10,11,12,13]. A study by Krachman et al. [14] evaluated 51 smokers with suspected OSA and reported an inverse relationship between emphysema severity and the apnea–hypopnea index (AHI), indicating that more advanced emphysema was associated with lower OSA severity. Physiologically, emphysema is associated with loss of elastic recoil, expiratory flow limitation, air trapping, and lung hyperinflation. The resulting increase in end-expiratory lung volume may augment caudal tracheal traction and stabilize the upper airway during sleep, thereby reducing pharyngeal collapsibility. In their CT-based analysis, greater CT-quantified emphysema and gas trapping were independently associated with lower AHI values, supporting a protective effect of lung inflation on OSA severity. By contrast, patients with COPD who have an obesity-predominant rather than emphysema-predominant phenotype typically exhibit less hyperinflation, a pattern that may favor OSA development. Schwartz et al. [15] demonstrated that reduced lung volume increases pharyngeal collapsibility and OSA risk, showing that obesity-related reductions in resting lung volume diminish caudal traction on upper airway structures and promote airway collapse.

Bronchiectasis and OSA may co-exist and a number of studies have reported a high prevalence of OSA in adults with non-cystic fibrosis bronchiectasis (NCFB). A Brazilian cross-sectional study involving 49 patients with NCFB detected OSA by overnight polysomnography in 40.8% of subjects. In addition, more than half of the patients were considered at high risk for OSA and experienced significant nocturnal oxygen desaturation [16]. Similarly, a study from Turkey including 43 individuals with NCFB reported an even higher prevalence of OSA at 55.8%. Most of these cases were classified as mild to moderate in severity, further indicating that OSA is a common comorbidity among patients with bronchiectasis [17]. The hypoxemia observed in bronchiectasis resembles the chronic intermittent hypoxia characteristic of OSA. In OSA, repeated cycles of hypoxemia followed by reoxygenation occur throughout the night, promoting oxidative stress and systemic inflammatory responses [18]. Biomarker studies have shown that patients with OSA often exhibit increased levels of total oxidative status, C-reactive protein, and transforming growth factor-β1, along with reduced levels of soluble receptors for advanced glycation end products. These changes indicate a shift toward a pro-inflammatory environment that tends to worsen as the severity of OSA increases [18].

In our study, the apparent higher risk of OSA among patients with emphysema in the unmatched analysis was substantially attenuated after propensity score matching, suggesting that the crude association was largely attributable to differences in baseline characteristics rather than emphysema itself. Before propensity score matching, patients with emphysema were more likely to be male (55.08% vs. 39.16%) and had higher prevalences of obesity, dyslipidemia, ischemic heart disease, and use of medications for nicotine dependence, several of which are also associated with OSA risk. Sex-related anatomical differences may further contribute to this imbalance, as recent evidence suggests that sex influences hyoid bone position and upper-airway anatomy in adults with OSA [19]. These baseline differences may therefore have contributed, at least in part, to the higher OSA risk observed in the unmatched emphysema cohort.

After 1:1 propensity score matching, sex distribution shifted dramatically: male proportion in the emphysema cohort dropped from 55.08% to 41.28%, nearly matching the bronchiectasis cohort (41.14%), because matching was constrained by the smaller, female-predominant bronchiectasis pool. Comorbidity differences also largely disappeared (Std diffs <0.05). Once these confounders were balanced, the direction reversed: bronchiectasis showed slightly higher short-term OSA risk, suggesting the pre-matching “emphysema effect” was largely driven by demographic and comorbidity imbalance rather than a genuine biological difference between the two diseases. These findings indicated that the higher OSA risk observed before matching was influenced, at least in part, by baseline differences between the two cohorts rather than by emphysema alone. After propensity score matching, the between-group difference became smaller over time, with HRs progressively approaching unity. In addition, the proportional hazards assumption was not met during longer follow-up, indicating that the relative hazard of OSA may change over time rather than remain constant throughout the study period. These findings suggest that the relative risk of OSA may vary over the course of follow-up and should be considered hypothesis-generating. Further prospective studies incorporating detailed pulmonary function, disease severity, and sleep-study data are needed to confirm these findings and clarify the underlying mechanisms.

This study has several limitations that should be acknowledged. First, the analysis was based on a retrospective dataset derived from the TriNetX research network. Diagnoses and clinical outcomes were identified primarily through diagnostic codes and clinical documentation without independent validation, which may introduce potential misclassification or inaccuracies in outcome identification. In addition, variations in coding practices, data entry errors, and differences in EHR systems across participating institutions may contribute to heterogeneity in the dataset and affect the overall reliability of the analyses. Second, detailed information on disease severity and clinical management was not consistently available in the database. Measures such as forced expiratory volume in 1 s, global initiative for chronic obstructive lung disease stage, bronchiectasis severity indices, CT-quantified emphysema extent, long-term oxygen therapy, healthcare utilization, and sleep-study use could therefore not be incorporated into the propensity score model. As a result, residual confounding and surveillance or detection bias may remain, particularly because differences in follow-up intensity and access to sleep testing could have influenced the likelihood and timing of OSA diagnosis. In addition, the absence of detailed physiological and imaging data prevented us from evaluating whether disease severity modified OSA risk. Accordingly, previously proposed mechanisms, including the inverse association between CT-quantified emphysema extent and AHI, cannot be directly tested or confirmed by the present study. Third, smoking data, including smoking intensity, duration, and cumulative pack-years, were unavailable. Although medications for nicotine dependence were included as a proxy, they may not fully reflect actual smoking behavior. Therefore, residual confounding from smoking exposure, disease severity, lifestyle factors, socioeconomic status, environmental exposures, and other unmeasured variables cannot be excluded. Accordingly, the findings should be interpreted as associations rather than causal relationships. Fourth, calendar year could not be incorporated into the propensity score matching model because of limitations of the TriNetX analytical platform. Therefore, potential temporal changes in the clinical recognition, diagnostic testing, and healthcare practices related to OSA could not be fully controlled, and residual calendar-time-related detection bias may remain. Despite these limitations, the large sample size and multicenter nature of the TriNetX network enhance the generalizability of our findings across diverse real-world healthcare settings. Although the results should be interpreted cautiously, they provide useful hypothesis-generating evidence and may help inform future studies examining the relationship between chronic structural lung disease and OSA.

## 5. Conclusions

After propensity score matching, there was no significant difference in the long-term hazard of OSA between patients with emphysema and those with bronchiectasis. Patients with bronchiectasis showed a modestly higher risk of OSA during the early follow-up period, but this difference gradually diminished over time. These findings suggest that the relative risk of OSA may vary during follow-up and should be considered hypothesis-generating. Further prospective studies incorporating detailed measures of disease severity, pulmonary function, and sleep-related parameters are needed to confirm these findings and clarify the underlying mechanisms.

## Figures and Tables

**Table 1:** Characteristics of emphysema and bronchiectasis cohorts before propensity score matching.

	Emphysema (n, %)	Bronchiectasis (n, %)	p-Value	Std Diff
Variables	(*n* = 526,877)	(*n* = 147,700)		
Age at index (mean ± SD)	64.0 ± 11.4	62.7 ± 14.7	<0.0001	0.0988
Sex				
Female	236,675 (44.92%)	89,864 (60.84%)	<0.0001	0.3231
Male	290,202 (55.08%)	57,836 (39.16%)	<0.0001	0.3231
Race				
White	378,231 (71.79%)	81,318 (55.06%)	<0.0001	0.3527
Black or African American	65,743 (12.48%)	13,334 (9.03%)	<0.0001	0.1115
Comorbidities				
Dyslipidemia	175,860 (33.38%)	35,205 (23.84%)	<0.0001	0.2123
Diabetes mellitus	78,247 (14.85%)	20,075 (13.59%)	<0.0001	0.0361
Ischemic heart disease	102,343 (19.43%)	18,332 (12.41%)	<0.0001	0.1926
Depression	75,250 (14.28%)	13,748 (9.31%)	<0.0001	0.1547
Chronic kidney disease	41,829 (7.94%)	10,669 (7.22%)	<0.0001	0.0270
Obesity	44,831 (8.50%)	10,121 (6.85%)	<0.0001	0.0622
Medication				
Glucocorticoids	188,280 (35.74%)	47,461 (32.13%)	<0.0001	0.0761
Statins	139,891 (26.55%)	25,600 (17.33%)	<0.0001	0.2241
Selective serotonin reuptake inhibitors	66,680 (12.66%)	12,877 (8.72%)	<0.0001	0.1277
Metformin	32,353 (6.14%)	7522 (5.09%)	<0.0001	0.0455
Drugs used in nicotine dependence	66,720 (12.66%)	3067 (2.08%)	<0.0001	0.4138

Std diff: standardized difference; SD: standard deviation.

**Table 2:** Characteristics of emphysema and bronchiectasis cohorts after propensity score matching.

	Emphysema (n, %)	Bronchiectasis (n, %)	p-Value	Std Diff
Variables	(*n* = 129,761)	(*n* = 129,761)		
Age at index (mean ± SD)	63.4 ± 13.6	62.6 ± 14.7	<0.0001	0.0540
Sex				
Female	76,190 (58.72%)	76,373 (58.86%)	0.4655	0.0029
Male	53,571 (41.28%)	53,388 (41.14%)	0.4655	0.0029
Race				
White	75,668 (58.31%)	76,424 (58.90%)	0.0026	0.0118
Black or African American	12,407 (9.56%)	13,297 (10.25%)	<0.0001	0.0230
Comorbidities				
Dyslipidemia	36,164 (27.87%)	34,679 (26.73%)	<0.0001	0.0257
Diabetes mellitus	19,094 (14.72%)	19,278 (14.86%)	0.3089	0.0040
Ischemic heart disease	18,958 (14.63%)	18,129 (13.97%)	<0.0001	0.0188
Depression	13,510 (10.41%)	13,581 (10.47%)	0.6485	0.0018
Chronic kidney disease	10,274 (7.92%)	10,247 (7.90%)	0.8443	0.0008
Obesity	10,502 (8.09%)	9945 (7.66%)	<0.0001	0.0159
Medication				
Glucocorticoids	45,165 (34.81%)	45,456 (35.03%)	0.2308	0.0047
Statins	26,696 (20.57%)	25,294 (19.49%)	<0.0001	0.0270
Selective serotonin reuptake inhibitors	12,729 (9.81%)	12,665 (9.76%)	0.6724	0.0017
Metformin	7137 (5.50%)	7255 (5.59%)	0.3115	0.0040
Drugs used in nicotine dependence	3165 (2.44%)	3067 (2.36%)	0.2089	0.0049

Std diff: standardized difference; SD: standard deviation.

**Table 3:** Risk of obstructive sleep apnea at 1-, 2-, 3-, 4-, and 5-year intervals, as well as cumulatively through 29 July 2026, before propensity score matching.

Period	Emphysema (n, Risk) (n = 526,877)	Bronchiectasis (n, Risk) (n = 147,700)	Risk Ratio (95% CI)	Odds Ratio (95% CI)	Hazard Ratio (95% CI)	p for Proportionality
1-year	8137 (1.544%)	2204 (1.492%)	1.035 (0.988, 1.048)	1.036 (0.988, 1.086)	1.033 (0.986, 1.083)	0.0826
2-year	13,189 (2.503%)	3459 (2.342%)	1.069 (1.030, 1.109)	1.071 (1.031, 1.112)	1.071 (1.031, 1.111)	0.2503
3-year	17,964 (3.358%)	4457 (3.018%)	1.113 (1.077, 1.149)	1.117 (1.080, 1.155)	1.119 (1.083, 1.156)	<0.0001
4-year	22,101 (4.195%)	5417 (3.668%)	1.144 (1.111, 1.178)	1.150 (1.116, 1.185)	1.155 (1.121, 1.190)	<0.0001
5-year	26,006 (4.936%)	6287 (4.257%)	1.160 (1.129, 1.191)	1.168 (1.135, 1.201)	1.177 (1.145, 1.210)	<0.0001
through 29 July 2026	36,266 (6.883%)	8703 (5.892%)	1.168 (1.142, 1.195)	1.181 (1.152, 1.209)	1.251 (1.222, 1.281)	<0.0001

CI: confidence intervals.

**Table 4:** Risk of obstructive sleep apnea at 1-, 2-, 3-, 4-, and 5-year intervals, as well as cumulatively through 29 July 2026, after propensity score matching.

Period	Emphysema (n, Risk) (n = 129,761)	Bronchiectasis (n, Risk) (n = 129,761)	Risk Ratio (95% CI)	Odds Ratio (95% CI)	Hazard Ratio (95% CI)	p for Proportionality
1-year	1734 (1.336%)	2140 (1.649%)	0.810 (0.761, 0.863)	0.808 (0.758, 0.861)	0.829 (0.778, 0.883)	0.0957
2-year	2889 (2.226%)	3356 (2.586%)	0.861 (0.820, 0.904)	0.858 (0.816, 0.902)	0.885 (0.843, 0.931)	0.1119
3-year	3886 (2.979%)	4328 (3.335%)	0.893 (0.856, 0.932)	0.890 (0.852, 0.930)	0.923 (0.884, 0.964)	0.0002
4-year	4785 (3.688%)	5256 (4.051%)	0.910 (0.876, 0.946)	0.907 (0.871, 0.944)	0.946 (0.910, 0.984)	<0.0001
5-year	5597 (4.313%)	6100 (4.701%)	0.918 (0.886, 0.951)	0.914 (0.881, 0.948)	0.960 (0.925, 0.995)	<0.0001
through 29 July 2026	7784 (5.999%)	8435 (6.500%)	0.923 (0.896, 0.951)	0.918 (0.889, 0.948)	1.006 (0.976, 1.038)	<0.0001

CI: confidence intervals.

## Data Availability

Data were obtained from the TriNetX platform (https://trinetx.com/), a global federated research network that integrates de-identified electronic health records from multiple healthcare institutions to support real-world clinical research.

## Abbreviations

The following abbreviations are used in this manuscript:

AHI	apnea–hypopnea index
ATC	Anatomical Therapeutic Chemical
CIs	confidence intervals
CKD	chronic kidney disease
COPD	chronic obstructive pulmonary disease
CT	computed tomography
DM	diabetes mellitus
EHR	electronic health record
HIPAA	Health Insurance Portability and Accountability Act
HRs	hazard ratios
ICD-10	International Classification of Diseases, Tenth Revision
NCFB	non-cystic fibrosis bronchiectasis
ORs	odds ratios
OSA	obstructive sleep apnea
RRs	risk ratios
Std diff	standardized differences
SSRIs	selective serotonin reuptake inhibitors
